# Solar inactivated *Salmonella* Typhimurium induces an immune response in BALB/c mice

**DOI:** 10.1016/j.heliyon.2021.e05903

**Published:** 2021-01-06

**Authors:** Cornelius C. Ssemakalu, Marta Ulaszewska, Sean Elias, Alexandra J. Spencer

**Affiliations:** a Cell Biology Research Unit, Department of Biotechnology, Faculty of Applied and Computer Sciences, Vaal University of Technology, Vanderbijlpark, 1911, South Africa; b The Jenner Institute, University of Oxford, Old Road Campus Research Building, Roosevelt Drive, Oxford, OX3 7DQ, United Kingdom

**Keywords:** SODIS, *S*. Typhimurium, Waterborne, Foodborne, Immunity and typhoid

## Abstract

*Salmonella* is contracted through the consumption of untreated water and contaminated food. The contraction and spread of water-related *Salmonella* in resource-poor communities can be reduced by using solar disinfection (SODIS) to treat the water before its consumption. SODIS is a water sanitizing technique that relies on natural sunshine. It is a cost-effective, inexpensive, environmentally, and user-friendly means of treating microbiologically contaminated water. This water disinfection method has saved many lives in communities vulnerable to water-related infections worldwide. At present, the success of SODIS has mainly been attributed to permanent inactivation of water pathogens ability to grow. However, little to no information exists as to whether immune responses to the solar inactivated pathogens are induced in SODIS water consumers. This study assessed the potential for solar inactivated *S*. Typhimurium to induce an immune response in mice. Results show that solar inactivated *S*. Typhimurium can induce bactericidal antibodies against *S*. Typhimurium. Furthermore, an increase in the secretion of interferon-gamma (IFN-γ) was observed in mice given the solar inactivated *S*. Typhimurium. These findings suggest that solar inactivated *S*. Typhimurium induces a humoral and cellular immune response. However, the level of protection afforded by these responses requires further investigation.

## 1 Introduction

*Salmonella enterica Serovar* Typhimurium a nontyphoidal salmonella (NTS) strain is the major cause of bacteremia in resource-poor communities in sub-Saharan Africa (SSA) and can have upto a 20 % fatality rate [1, 2]. Deaths due to NTS are prevalent among children between the age of 6 months and 5 years, and those suffering from the acquired immunodeficiency syndrome or malaria [3]. Furthermore, several clinically relevant NTS isolates in SSA have developed antimicrobial resistance against various antibiotics, thus limiting treatment options [1, 4]. *Salmonella* Typhimurium is mainly contracted through the consumption of untreated water and contaminated food [2, 3, 5]. The contraction and spread of waterborne pathogens such as NTS could be prevented through proper sanitation and hygiene and the use of treated water for domestic purposes. Although there has been a reduction in deaths due to water-related infections, the lack of safe drinking water remains a significant impediment to sustainable livelihood and economic development in SSA [6]. Solar disinfection (SODIS) is a cost-effective, inexpensive, environmentally and user-friendly means of treating microbiologically contaminated water that has saved lives of people vulnerable to water-related infections worldwide [7, 8, 9, 10]. The SODIS technique requires little to no cost because it relies on a transparent clear vessel and natural sunshine. The alternative means of disinfecting water available to people in resource poor communities such as boiling of water and chlorination require some financial input. The ability of SODIS to inactivate waterborne pathogens, including *S.* Typhimurium, has been attributed to the deleterious effects derived from solar ultraviolet radiation (SUVR) particularly UVA and also the synergy between SUVR and temperature [11]. SUVR has been reported to sterilise microbiologically contaminated water through the formation of reactive oxygen radicals that cause oxidative stress to water contaminating microorganisms [12]. Various laboratory and field experiments have shown that SUVR causes permanent inactivation of *S.* Typhimurium without any regrowth following dark storage periods [13]. Furthermore, *S.* Typhimurium exposed to SODIS treatment was found to have lost infectivity [14]. To date, much effort has been placed on understanding the mechanisms through which microbial inactivation occurs [12, 15]; scaling up of the technology [16, 17]; and epidemiological impact [7, 9, 10]. Little to no information exists as to the impact of irradiated solar pathogens on the immunity of the consumers [18]. Following SODIS treatment, the dead microbial cells and debris exist in various states [19, 20]. However, the immunogenicity of these microbial cells, as well as debris, remains mostly unknown, thus leaving a significant gap in SODIS research. As such, there remain some unanswered questions; the most important being whether consumers of SODIS water containing waterborne pathogens, such as NTS, derive an immunological benefit or not. The idea of SODIS linked immune benefits has been reviewed elsewhere [18]. In essence, it is crucial to take into account the quantity, quality and rate of appearance of antigens [21] generated during SODIS. The current dogma dictates that high antigen dose is required to reduce the chance of producing antigen-specific regulatory cells [22]. During an outbreak, the bacterial load of *Salmonella* is sufficient enough to cause disease. The SODIS method has been shown to inactivate bacterial amounts greater than the number of *Salmonella* bacterial cells needed to cause an infection. Besides the antigen dose, it is vital to consider the possibility that SODIS could cause sufficient immunity to control infections in people living in waterborne disease endemic communities that rely on this technique to sterilise their water. Also, the potential for SODIS treatment to induce slow or extreme rapid modifications of the antigenic epitopes remains unknown. Therefore, there is a need to explore the immunogenicity of SODIS inactivated microorganisms. In this study, the potential for a solar irradiated NTS isolate to induce a humoral or cellular immune response in a mouse model was investigated. Both humoral and cellular responses have been shown to play a vital role in the protection and prevention of infection resulting from NTS isolates in the mouse model [23, 24].

## 2 Materials and methods

### 2.1 Bacterial culture preparation

*Salmonella enterica Serovar* Typhimurium ST313 strain D23580 was inoculated on to Luria Betarni (LB) agar from a bead stock and the plates incubated at 37 °C overnight. A single colony of *S.* Typhimurium was then transferred into LB broth and grown at 37 °C for 18 h until it reached the stationary phase. Bacterial cells at the stationary phase were used because they are resilient to ultraviolet treatment [25, 26].

### 2.2 Bacterial preparation prior and enumeration after SUVR exposures

An overnight culture of *S.* Typhimurium was harvested by centrifugation at 1600 g for 10 min and washed three times with ultra-pure water. The pellet was resuspended in ultrapure water to an optical density (OD600) of 0.24 corresponding to 9 Log Colony Forming Units per ml (Log CFU/ml) or ~5 x 10^8^ CFU/ml. 100 ml of the cell suspension was then transferred into 150 cm^3^ tissue culture flasks, vigorously shaken and then exposed to solar irradiation over aluminium foil under atmospheric conditions for 4, 6 and 8 h. Control samples were exposed to similar climatic conditions except for SUVR, that was eliminated by covering the control samples with an opaque ventilated box. Solar exposures were performed on an unshaded rooftop (Latitude 51.752612 and Longitude -1.214508). Solar ultraviolet irradiation was recorded by the Bentham DM150 spectroradiometer located at the Reading University Atmospheric Observatory [27]. The air temperature data were obtained from the weather observation site located within a 5 km radius from the point of exposure [28]. After each time point, the solar irradiated and corresponding non-solar irradiated samples were retrieved and kept in the dark at room temperature (22 °C) overnight. The viability of *S.* Typhimurium in both the solar and non-solar irradiated samples was then assessed using the Miles and Misra drop method [29] with modifications. In brief, 10 μl of the appropriate dilution was dropped onto sterile LB agar plates in quadruplicate. The plates were then incubated at 37 °C overnight, and plates with less than 50 discrete colonies per drop were selected and counted. The total count was divided by the number of drops, multiplied by 100 to convert to 1 ml, and then divided by the dilution factor to give the number of CFU/ml [20]. The samples were kept at 4 °C while viability was being assessed.

### 2.3 Preparation of solar inactivated *Salmonella* Typhimurium vaccine

Only samples where total inactivation in the viability of *S.* Typhimurium had occurred through SUVR exposure were used for the animal study. The solar inactivated culture of *S.* Typhimurium was harvested by centrifugation at 6500 g for 20 min and resuspended to a final concentration of 1 x 10^10^ CFU/ml in PBS. The solar inactivated *S.* Typhimurium vaccine was stored at -80 °C until it was needed.

### 2.4 Ethics statement

Mice were used in accordance with the UK Animals (Scientific Procedures) Act under project license number P9804B4F1 granted by the UK Home Office and reviewed by the University of Oxford Animal Care and Ethical Review Committee. Animals were group housed in IVCs under SPF conditions, with constant temperature and humidity with lighting on a 12:12 light-dark cycle (8am to 8pm). All animals were humanely sacrificed at the end of each experiment by an approved Schedule 1 method.

### 2.5 Immunization and sample collection

Female 5–6 weeks old BALB/c mice were divided into two groups of six mice each and acclimatized for one week before experiments began. Throughout the study food and water was given ad libitum. The experimental group was primed with 1 x 10^9^ bacterial cells of the solar inactivated *S.* Typhimurium in 100 μl of PBS by oral gavage. The control group was administered with 100 μl of PBS by oral gavage. The mice were then given boosters of the solar inactivated *S.* Typhimurium at days 14 and 28 post-prime inoculation (PPI). A blood sample from each mouse in both the experimental and control groups was collected from the tail vein after 21 days PPI. Faecal samples were collected after 11, 24, 33 and 42 days PPI. After 42 days PPI, the mice in each group were sacrificed, with blood and spleens collected for analysis.

### 2.6 Preparation of serum and faecal samples

Blood samples were kept at 4 °C overnight and centrifuged at 13,500 g for 5 min. The resultant serum was then stored at -20 °C until it was needed for either the Enzyme-Linked Immunosorbent Assay (ELISA) or Serum Bactericidal Assay (SBA). The faecal pellets were prepared for the ELISA as described elsewhere [30] with some modifications. Supernatants from the faecal pellets were prepared by adding 1ml of the faecal extraction buffer (FEB) consisting of PBS and 1% Protease inhibitor (Cat No: 87786, Thermo Fisher Scientific, MA) to 100 mg of the faecal sample. Following the addition of FEB, the samples were placed in the fridge at 4 °C for 10 min. Samples were briefly vortexed and further homogenized using the Eppendorf thermomixer comfort (Hamburg, Germany) platform shaker operated at 200 g, 6 °C for 40 min. The samples were kept at 4 °C for 20 min and then centrifuged at 13,500 g for 10 min at 4 °C. The supernatant was then stored at -20 °C until required.

### 2.7 Serum IgG and faecal IgA antibody ELISA

An endpoint ELISA was performed to determine serum IgG antibody titers against the whole bacterial cell (BACT) of *S.* Typhimurium strain D23580, as well as the commercially available LPS (ALX-581-011-L002) and FliC (SRP 8029, Sigma, MO) from *S.* Typhimurium. Both LPS and FliC were prepared in carbonate buffer (Sigma C-3041) and used for ELISA at a concentration of 5 μg/ml and 100 ng/ml respectively. Faecal IgA antibody titers were determined against the BACT of *S.* Typhimurium strain D23580 only. The BACT ELISA was done as described elsewhere [31] with modifications. An overnight culture of *S.* Typhimurium was harvested by centrifugation at 2200 g for 10 min washed with PBS and eventually resuspended in coating buffer to an optical density (OD600) of ~0.2 corresponding to 8 Log CFU/ml or ~8 x 10^7^ CFU/ml. The overnight culture was then placed in a water bath set at 56 °C for 1 h. After that a 96-well Maxisorb Microtiter ELISA plate was coated with 50 μl/well of the heat-treated overnight culture of *S.* Typhimurium, LPS or FliC and then incubated at 4 °C overnight. The plate was then washed five times with wash buffer (PBS with 0.05% Tween 20), and 100 μl of blocking buffer (1% BSA in PBS) was added followed by a 2-hour incubation at room temperature. Plates were washed five times, and either the faecal supernatant or serum was added. The faecal supernatant was added and diluted two-fold from 1 in 3 in blocking buffer. The serum was added and diluted two-fold starting from a dilution of 1in 50 in blocking buffer. Blocking buffer was used as the blank while anti*-S.* Typhimurium – LPS antibody (ab 8274, Abcam, Cambridge, United Kingdom) 1: 20000 was used as a positive control. The plate was then incubated at room temperature for 2 h, washed five times with wash buffer and then 50 μl of either horseradish peroxidase (HRP) conjugated goat anti-mouse IgA antibody (Cat No: 62-6720, Thermo Fisher Scientific, MA) (at 1 in 1000) or HRP conjugated goat anti-mouse IgG antibody (Cat No: 31430, Thermo Fisher Scientific, MA) (at 1 in 5000) was added. After 90 min of incubation at room temperature, plates were washed five times, and TMB substrate (Cat No: 34021, Thermo Fisher Scientific, MA) added. The plates were allowed to develop for 15 min (IgG) or 30 min (IgA). Then 2 M sulphuric acid was added to stop the reaction, and the plate was read at 450 nm (EL-800, BioTek, VT) within 30 min.

### 2.8 Serum bactericidal activity assay

A single colony of *S.* Typhimurium strain D23580 was inoculated into 10 ml of LB broth and grown overnight (18 h) at 37 °C. The overnight culture was diluted 1000-fold in fresh LB broth and incubated at 37 °C. After 2 h of incubation, 3 ml was centrifuged at 8000 g for 5 min and then washed twice with PBS. The bacterial cells were then resuspended in PBS to a final OD_600nm_ of ~0.2, diluted 10000-fold and used for the serum bactericidal assay. A titration to determine the number of bacteria cells was done and showed that ~1000 cells had been added. An aliquot of serum collected at day 42 was incubated at 56 °C for 30 min to inactivate endogenous complement. After that, bacteria were added to the heat-inactivated serum supplemented with baby rabbit complement (CL3441, Cedarlane, Burlington, Canada) and then incubated at 37 °C. After 1 h, bacterial viability was assessed using the Miles and Misra drop method [29] with modifications. In brief, 10 μl of the appropriate dilution was dropped onto sterile LB agar plates in quadruplicate. The plates were then incubated at 37 °C overnight, and plates with less than 50 discrete colonies per drop were selected and counted. The total count was divided by the number of drops, multiplied by 100 to convert to 1 ml, and then divided by the dilution factor to give the number of CFU/ml [20]. Complement, PBS and heat-inactivated serum without complement served as controls. The percentage SBA was calculated using Eq. (1).

(1) $$\%\;SBA=100-\left(\frac{CFU/ml\;of\;the\;sample}{CFU/ml\;of\;Compliment}\right)\;x\;100$$

### 2.9 Isolation of spleen lymphocytes

Single cell suspensions of splenocytes were prepared by manual crushing of spleens and passing suspensions through a 70 μm sieve, and the filtrate was centrifuged at 600 g for 5 min. To remove red blood cells, Ammonium-Chloride-Potassium lysis buffer was added for 5 min prior to washing with PBS and centrifugation at 600 g for 5 min. The cells were then resuspended in complete culture media consisting of α-MEM media supplemented with 10% fetal calf serum (Sigma F-2442, Merck), 1% pen/strep (Sigma P-0781), 1% L-glutamine [4mM] (Sigma G-7513, Merck) and 0.1% 2-Mercaptoethanol (BRL 31350-010, Gibco), counted and used for the IFN-γ Elispot assay and lymphocyte characterization.

### 2.10 IFN-γ ELISpot assay

Multiscreen IP filter plates (MAIPS4510, Merck) were coated with an IFN-γ antibody (Clone AN18 (Mabtech, Stockholm, Sweden)) prepared at a concentration of 5 μg/ml in carbonate-bicarbonate buffer. Following removal of coating antibody and blocking of plates, splenocytes (1 x 10^7^ cells/ml) were added and serially diluted in complete culture media. Stimulants LPS, FliC and BACT, prepared in complete culture media were added at concentrations of 1 μg/ml, 0.5 μg/ml and 1 x 10^8^ CFU/ml and the plates were incubated at 37 °C overnight. After that, the plates were washed six times with PBS and then biotinylated rat anti-mouse interferon-gamma (Clone R4-6A2) (Mabtech) diluted to 1 μg/ml in PBS was added. The plates were incubated for 2 h at room temperature, prior to washing and addition of Streptavidin Alkaline Phosphatase Polymer (Mabtech) diluted to 1 μg/ml in PBS. Following incubation for at least 1 h at room temperature and washing six times with PBS, ELISpot colour development solution (170-6432, Bio-Rad, CA) was added. The plates were incubated at room temperature until spots developed and then the development solution was washed off with tap water. The spots were then counted with an ELISpot reader (AID ispot, Straßberg, Germany) and the number of spot forming splenocytes calculated.

### 2.11 Characterisation of the lymphocytes

Splenocytes (1 x 10^7^ cells/ml) in a round bottom 96 well plate were centrifuged at 800 g for 3 min and the supernatant discarded. The cell pellet was then resuspended in PBS-BSA, centrifuged at 800 g for 3 min and the supernatant discarded. Cells were resuspended in a cocktail consisting of an aqua-live/dead cell marker (Invitrogen) as well as mouse antibodies against cell surface markers CD80-FITC, γδ-PerCPCy710, F4/80-e450, CD8-BV650, CD4-BV711, CD11c-A647, I-A/I-E-A700, CD11b-APCA780, DX5-PE and B200-PECy7 diluted in PBS-BSA and incubated for at least 30 min at 4 °C. Following addition of PBS-BSA, cells were then centrifuged at 800 g for 3 min, the supernatant discarded, cells were resuspended in PBS/BSA for acquisition on a flow cytometer (LSR Fortessa X-20, Becton Dickinson, NJ).

### 2.12 Statistical analysis

The animal data were expressed as individual data points with lines depicting the median and error bars denoting the interquartile range. All other data was expressed as means ± standard errors. To define statistically significant differences, the data were analysed with one-way analysis of variance (ANOVA) assuming equal variances at a p < 0.05 level unless stated otherwise.

## 3 Results

### 3.1 Solar inactivation of *S.* Typhimurium

**Figure 1:**
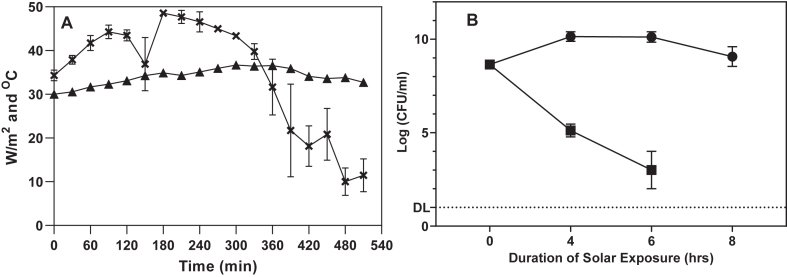
A) Solar irradiance (x) and temperature (black triangles) recorded every 30 min over the 8 h of solar irradiation. (B) Assessment of the viability of solar (black squares) and non-solar (black circles) irradiated *S*. Typhimurium. Error bars indicate SEM of duplicated experiments done on different days. DL = Detection limit.

Solar exposures were carried out from 0900H to 1700H on two sunny days. During this period the solar irradiance varied in the range between 48 to 10 W/m^2^ (34.63 ± 2.99 W/m^2^) while the temperature fluctuated in the range between 36 and 30 °C (34 ± 0.47 °C) (Figure 1A). On both days, the viability of the solar irradiated *S.* Typhimurium reduced until it was no longer culturable after 8 h (Figure 1B). The mean solar radiant fluence after 8 h of exposure for both days was 1080.86 ± 61.29 kJ/m^2^.

### 3.2 Serum IgG and fecal IgA ELISA

**Figure 2:**
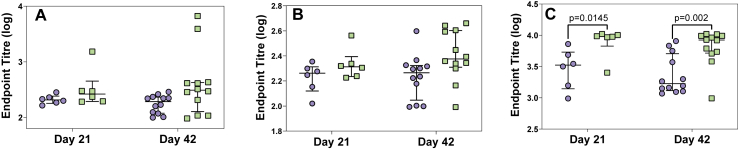
Serum IgG antibody responses against *S*. Typhimurium (A) LPS, (B) FliC and (C) BACT. Each data point represents an individual mouse (from 1 experiment (Day 21) and 2 separate experiments (Day 42)) that was either given PBS (blue) or Solar Inactivated *S*. Typhimurium (green). Lines represent the median, with error bars denoting the interquartile range. Data in each graph was analysed with a 2-way ANOVA and post-hoc positive test, p values denote significance between vaccination groups at each time point.

**Figure 3:**
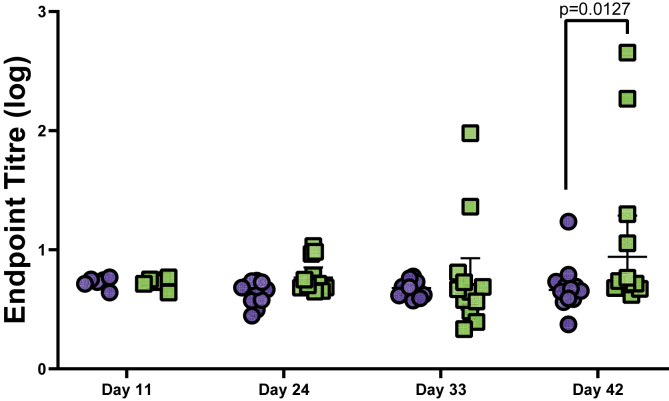
Fecal IgA antibody responses against the whole bacterial cell of *S*. Typhimurium. Each data point represents an individual mouse (from 1 experiment (Day 11) and 2 separate experiments (Days 24, 33 and 42)) that was either given PBS (blue) or Solar Inactivated *S*. Typhimurium (green). Lines represent the median, with error bars denoting the interquartile range. Data was analysed with a 2-way ANOVA and post-hoc positive test, p values denote significance between vaccination groups at each time point.

After administering treatment (solar inactivated *S*. Typhimurium or PBS), no weight loss was observed in either the SODIS treated or PBS treated mice. Serum from mice in both the experimental and control groups was used to assess IgG antibody responses to LPS, FliC and BACT. After 21 and 42 days, PPI a slight, albeit not statistically significant, increase in IgG antibodies against LPS and FliC was observed in the experimental group in comparison to the control (Figure 2A and B). Taking both time points together, analysis by 2-way ANOVA demonstrated a significant effect of vaccination in IgG responses to LPS (*p* = 0.0371) and FliC (*p* = 0.0313). In addition, a significant increase in serum IgG antibody response against the whole bacterial cell (BACT) was observed (p = 0.0005) in mice administered with solar inactivated treated S. Typhimurium, with significant difference between IgG levels observed at both 21 (*p* = 0.0145) and 42 (*p* = 0.002) days PPI (Figure 2C), but no difference between 21 and 42 days PPI in the experimental group was observed (p = 0.345). Faecal IgA antibody titres were measured against BACT after 11, 24, 33 and 42 days post-prime inoculation. The data demonstrated an increase in IgA antibodies in faecal samples against BACT in mice receiving solar inactivated S. Typhimurium (*p* = 0.0306), with a significant increase (Figure 3) observed after 42 days (*p* = 0.0127; treated vs control) post-prime inoculation.

### 3.3 Serum bactericidal assay

**Figure 4:**
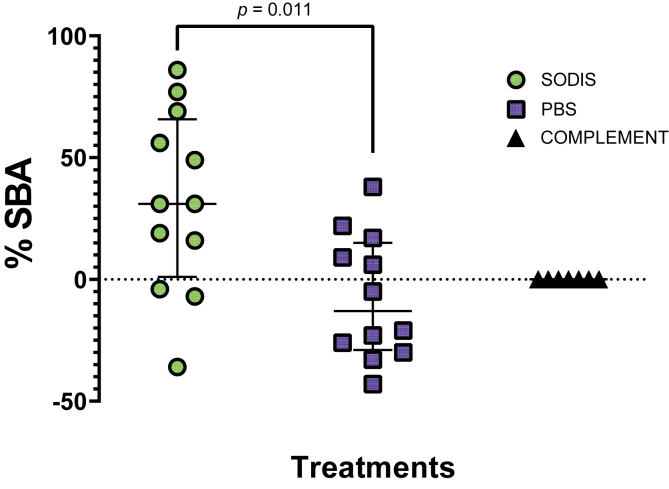
Serum bacterial Assay showing the percentage growth inhibition of *S*. Typhimurium following treatment with serum from mice vaccinated with either solar inactivated *S*. Typhimurium (green circles), or PBS (blue squares) and as a control complement (black triangles). Each green and blue data point represents serum from an individual mouse (from 2 separate experiments (Day 42)). Lines represent the median, with error bars denoting the interquartile range. Percentage inhibition was calculated relative to the treatment with complement only. Therefore, 0% inhibition would mean that there was no observed inhibition in the growth of *S*. Typhimurium. Any percentage value over 0% would indicate the magnitude of bacterial growth inhibition. Any percentage value below 0% would mean the extent of bacterial growth and hence the lack bacterial growth inhibition.

The ability of serum to inhibit the growth of *S*. Typhimurium was assessed using a serum bactericidal assay. The results (Figure 4) were expressed as the percentage reduction in the growth of *S*. Typhimurium following treatment with serum from mice vaccinated with either the solar inactivated *S*. Typhimurium or PBS. When *S*. Typhimurium was exposed to serum from mice vaccinated with solar inactivated *S*. Typhimurium a 32.25 ± 10.68% growth inhibition was observed. The growth of *S*. Typhimurium treated with serum from mice given PBS was not inhibited (- 7.4 ± 7.38%). An unpaired Mann-Whitney t-test revealed a significant (*p* = 0.0116) difference in the mean percentage growth inhibition of *S*. Typhimurium between serum derived from mice vaccinated with either the solar inactivated *S*. Typhimurium or PBS (Figure 4).

### 3.4 IFN-γ ELISpot assay

**Figure 5:**
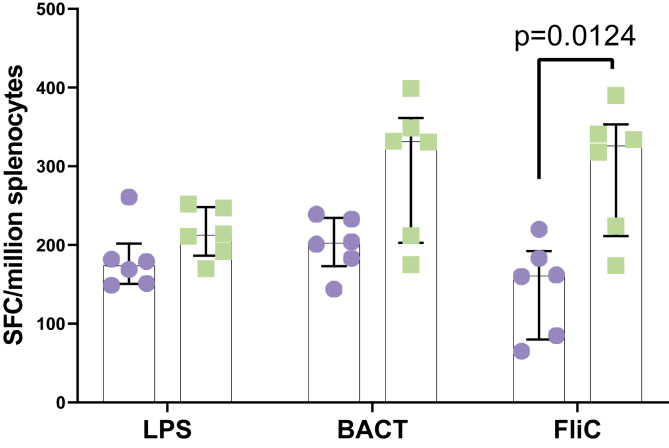
IFN-γ ELISpot assay showing the number of spot forming splenocytes in mice administered with either solar inactivated *S*. Typhimurium (green), or PBS (blue). LPS, FliC and BACT, were used as stimulants. Each data point (circle or square) represents an individual mouse, (from 1 experiment (Day 42)) and the error bars denote the interquartile range of the median. Data was analysed with a 2-way analysis of variance and post-hoc positive test, p values indicate significant differences between treatments groups for individual antigens.

The ability for the solar inactivated *S*. Typhimurium to induce the production of IFN-γ by splenocytes in response to LPS, FliC and BACT was assessed using an IFN-γ ELISpot assay. The number of IFN-γ secreting splenocytes observed in mice administered with solar irradiated *S*. Typhimurium was significantly higher than that observed in mice administered with PBS (Figure 5). A significant difference (*p* = 0.0124) in the number of IFN-γ secreting cells in response to FliC observed in splenocytes from mice vaccinated with solar irradiated *S*. Typhimurium was higher than that observed mice given PBS (Figure 5). A 2-way ANOVA and post-hoc positive test revealed a significant effect of vaccination in IFN-γ responses (*p* = 0.0079), with a significant increase in the number of FLiC specific IFN-γ producing splenocytes (p = 0.0124).

### 3.5 Characterization of the lymphocytes

**Figure 6:**
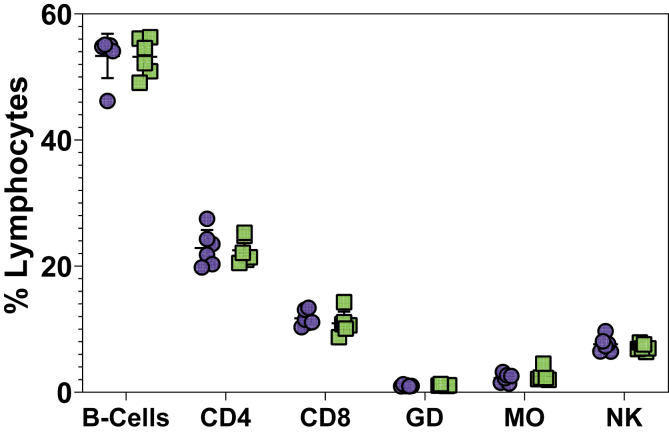
Percentage of B-Cells, CD4, CD8 and Gamma-Delta (GD) T-cells, Macrophages (MO) and Natural Killer (NK) cells in the spleens. Each data point represents an individual mouse (from 1 experiment (Day 42)) that was either given PBS (blue) or Solar Inactivated *S.* Typhimurium (green). Lines represent the median, with error bars denoting the interquartile range.

The number of the different lymphocytes in the spleens of mice either vaccinated with solar irradiated *S*. Typhimurium or given PBS was evaluated. Results showed a high presence of B-cells, as well as CD4 and CD8 T-cells in the spleens of mice regardless of treatment (Figure 6). No significant difference in the number of lymphocytes was observed.

## 4 Discussion

Solar irradiation of waterborne pathogens is an effective means of sterilizing water before its consumption. However, can solar inactivated waterborne pathogens confer any immunological benefits? This study investigated the potential for a solar inactivated NTS isolate to induce a humoral or cellular immune response in a mouse model. To achieve the aim of the study, there was a need to inactivate the viability of an NTS isolate using solar irradiation. Results from this study show that solar radiation can inactivate the viability of *S.* Typhimurium without regrowth following 18 h of dark storage. This observation confirms previously published data showing the ability of solar irradiation to inactivate the growth of *S.* Typhimurium [32]. The solar inactivated *S.* Typhimurium was then given to BALB/c mice and immune responses measured. BALB/c mice were used because they are susceptible to *S.* Typhimurium infection and hence an ideal animal model [23].

Upon the administration of the solar inactivated *S.* Typhimurium, the mice were observed throughout the entire duration of the experiment for any signs of ill health such as weight loss, the lack of appetite and reduced inactivity. The welfare charts showed that the mice did not lose any weight; furthermore, they did not show any signs of loss of appetite or loose stool. These observations indicate that the mice fed solar inactivated *S.* Typhimurium were as healthy as those given PBS. This suggests that solar inactivated *S.* Typhimurium is indeed no longer infectious and may not have resulted in the production of toxins during the inactivation process that could have contributed to the detrimental health of the mice. Similar observations have been previously reported [14].

To determine whether there was an immunological benefit in the consumption of solar inactivated waterborne pathogens antibody production in mice given the solar inactivated *S.* Typhimurium was assessed. It is important to note that this study used the oral route when administering the solar inactivated *S.* Typhimurium as well as PBS to the mice. Blood and faecal samples from mice given either solar inactivated *S.* Typhimurium or PBS were assessed for antibody production in response to LPS, FLIC and BACT. The results showed that mice fed solar inactivated *S.* Typhimurium overall had significantly (*p* = 0.0005) higher antibody responses compared to mice given PBS. This observation suggests that solar inactivated *S.* Typhimurium is capable of inducing the production of antibodies with the ability to recognize key antigenic determinants such LPS and FLIC of *S.* Typhimurium. The highest levels of antibodies measured were observed against whole bacteria as opposed to single antigens LPS or FLIC. Thus suggesting that antibodies produced may recognize a breadth of antigens in addition to LPS and FLIC. The results also showed that there was no significant (p = 0.345) increase in antibody response to BACT between 21 and 42 days. This suggests that the level of antibody response was neither enhanced or marred by subsequent administration of solar inactivated *S.* Typhimurium. Antibody production is the hallmark of a humoral immune response. This means that epitopes on solar inactivated *S.* Typhimurium not only survived the harsh oxidation processes but they also survived the gastric juice found in the stomach. Furthermore, epitopes from the solar inactivated *S.* Typhimurium were capable of being processed by antigen-presenting cells with the subsequent presentation to T-cells and eventually to B-cells.

Although antibody production is an essential indicator of immunogenicity, it was necessary to determine if the secreted antibodies were functional [33]. A serum bactericidal assay was used to determine if the secreted antibodies were capable of inhibiting the growth of viable *S.* Typhimurium in a complement-dependent manner. Results showed that serum antibodies produced by mice fed solar inactivated *S.* Typhimurium significantly (p = 0.011) inhibited the growth of *S.* Typhimurium in comparison to mice fed PBS. This observation suggests that the solar inactivated *S.* Typhimurium can induce antibodies that act in a complement dependent manner. Complement activation is one of the ways through which antibodies can cause the destruction of pathogenic microorganisms in the body. Activation of the complex by antibodies results in the formation of a membrane attack complex which confers irreversible and detrimental damage to the cell membrane tagged by antibodies [33]. Perhaps some of the antibodies could have had a bacterial static effect or result in the agglutination of the cells of *S.* Typhimurium. Alternatively, the antibodies could have had opsonizing capabilities, thus enhancing phagocytic activity of phagocytes. Although these eventualities were possible fates in antibody response, they were not investigated in the current study and may need to be explored further.

Besides the humoral response, cellular responses characterized by interferon-gamma secretion was assessed. The results showed that there was a significant (*p* = 0.0079) increase in IFN-γ production in mice given solar inactivated *S.* Typhimurium in comparison to those given PBS. Furthermore, the IFN-γ response to FliC was stronger in comparison to LPS or BACT. This means that solar inactivated *S.* Typhimurium was capable of inducing an IFN-γ response. IFN-γ production has been used as a measure of a cellular immune response. This protein is produced by T-cells in response to a previously recognized epitope. However, it should be noted that the IFN-γ in this study was measured in splenocytes. Splenocytes are a collective of cells consisting of a diverse population of white blood cells isolated from the spleens. As such, the measured IFN-γ response could have been due to either T-cells or Natural killer cells, among other IFN-γ secreting cells [34]. Nonetheless, IFN-γ secretion in moderation is a desirable immunological event that has been linked to the availability of immunological memory [34]. These results suggest that solar inactivated *S.* Typhimurium is not only able to evoke a humoral immune response but also a cellular immune response. An assessment of changes in immune cells in the spleen following administration of solar inactivated S. Typhimurium or PBS was also performed. The results showed that there was a high abundance of B-Cells and T-Cells. However, the difference in the mean percentage number per cell type between the spleens from mice given solar inactivated *S.* Typhimurium or PBS was not significant.

In conclusion, the findings of this study suggest that solar inactivated *S.* Typhimurium confer some immunological benefits to SODIS users, albeit overall low levels of antibodies and IFN- γ specific responses. However, it is critical to consider perhaps when this may be possible because a sufficient amount of antigen load may be required for a beneficial immune response to occur. For instance, the infectious dose of *S.* Typhimurium ranges from 10^3^ to 10^9^ [35]. In the absence of a *S.* Typhimurium linked waterborne outbreak, the bacterial load of this pathogen is probably lower than the reported infectious dose. Solar irradiation will inactivate the growth of water containing *S.* Typhimurium at doses below the infective dose. But it is highly unlikely that there might be an immunological benefit in consuming the solar inactivated pathogens at such a low dose because of a low antigen dose. Nonetheless, before and or during *salmonella* linked waterborne outbreak, the bacterial load increases. So probably the potential immunological benefit of consuming solar disinfected water could be beneficial then. Nevertheless, SODIS users are encouraged to use this water disinfecting method continuously whether or not there is an outbreak. Further work is planned to assess alternative routes of administration and the effect of dose.

## Declarations

### Author contribution statement

Cornelius C Ssemakalu, Alexandra J Spencer: Conceived and designed the experiments; Performed the experiments; Analyzed and interpreted the data; Contributed reagents, materials, analysis tools or data; Wrote the paper.

Marta Ulaszewska: Performed the experiments; Analyzed and interpreted the data.

Sean Elias: Conceived and designed the experiments; Analyzed and interpreted the data; Contributed reagents, materials, analysis tools or data; Wrote the paper.

### Funding statement

This work was supported by the African Research Excellence Fund [grant number MRF-157-0033-F-SSEMA(A)/C0751].

### Data availability statement

Data will be made available on request.

### Declaration of interests statement

The authors declare no conflict of interest.

### Additional information

No additional information is available for this paper.
